# Activation of p53 with Ilimaquinone and Ethylsmenoquinone, Marine Sponge Metabolites, Induces Apoptosis and Autophagy in Colon Cancer Cells

**DOI:** 10.3390/md13010543

**Published:** 2015-01-16

**Authors:** Hyun-Young Lee, Kyu Jin Chung, In Hyun Hwang, Jungsuk Gwak, Seoyoung Park, Bong Gun Ju, Eunju Yun, Dong-Eun Kim, Young-Hwa Chung, MinKyun Na, Gyu-Yong Song, Sangtaek Oh

**Affiliations:** 1 Department of Bio and Fermentation Convergence Technology, Kookmin University, Seoul 136-702, Korea; E-Mails: IHyunYoungg@gmail.com (H-Y.L.); qkr092@nate.com (S.P.); 2 College of Pharmacy, Chungnam national University, 305-764 Daejeon, Korea; E-Mails: doccap@naver.com (K.J.C.); yunej@cnu.ac.kr (E.Y.); 3 Department of Chemistry, University of Iowa, Iowa City, IA 52242, USA; E-Mail: inhyun-hwang@uiowa.edu; 4 Department of Life Science, Sogang University, Seoul 121-742, Korea; E-Mails: dmulder@dreamwiz.com (J.G.); bgju@sogang.ac.kr (B.G.J.); 5 Department of Bioscience and Biotechnology, Konkuk University, Seoul 143-701, Korea; E-Mail: kimde@konkuk.ac.kr; 6 BK21+, Department of Cogno-Mechatronics Engineering, Pusan National University, Busan 609-735, Korea; E-Mail: younghc@pusan.ac.kr

**Keywords:** ilimaquinone, ethylsmenoquinone, p53, colon cancer, apoptosis, autophagy

## Abstract

The tumor suppressor, p53, plays an essential role in the cellular response to stress through regulating the expression of genes involved in cell cycle arrest, apoptosis and autophagy. Here, we used a cell-based reporter system for the detection of p53 response transcription to identify the marine sponge metabolites, ilimaquinone and ethylsmenoquinone, as activators of the p53 pathway. We demonstrated that ilimaquinone and ethylsmenoquinone efficiently stabilize the p53 protein through promotion of p53 phosphorylation at Ser15 in both HCT116 and RKO colon cancer cells. Moreover, both compounds upregulate the expression of p21*^WAF1^*^/*CIP1*^, a p53-dependent gene, and suppress proliferation of colon cancer cells. In addition, ilimaquinone and ethylsmenoquinone induced G_2_/M cell cycle arrest and increased caspase-3 cleavage and the population of cells that positively stained with Annexin V-FITC, both of which are typical biochemical markers of apoptosis. Furthermore, autophagy was elicited by both compounds, as indicated by microtubule-associated protein 1 light chain 3 (LC3) puncta formations and LC3-II turnover in HCT116 cells. Our findings suggest that ilimaquinone and ethylsmenoquinone exert their anti-cancer activity by activation of the p53 pathway and may have significant potential as chemo-preventive and therapeutic agents for human colon cancer.

## 1. Introduction

The tumor suppressor, p53, plays an important role in maintaining cellular homeostasis in response to a wide variety of stresses, including DNA damage, activated oncogenes and various metabolic changes [1,2]. A loss of p53 function, either through direct mutations in the *p53* gene or indirect alterations in p53 regulatory networks, is frequently observed in human cancer [3] and is associated with a high rate of genomic instability, rapid tumor progression, resistance to anti-cancer therapy and increased angiogenesis [4,5]. In the absence of stress, the p53 protein is recognized by the E3 ubiquitin ligase, MDM2, which leads to its ubiquitination and degradation [6]. In response to cellular stress, the p53 protein experiences post-translational modifications, such as phosphorylation and acetylation [7,8], which allow for its accumulation and translocation into the nucleus, where it activates target genes involved in cell cycle arrest, apoptosis, senescence, anti-angiogenesis and autophagy, thereby suppressing malignant tumor transformation and preserving genomic integrity [9,10].

Ilimaquinone, a sesquiterpene quinone compound, was originally isolated from the Hawaiian sponge, *Hippospongia metachromia* [11], and boasts a number of biological studies. As a result, this compound is known to promote fragmentation of the Golgi apparatus through a microtubule-independent mechanism, thereby inhibiting vesicular protein transport [12]. In addition, ilimaquinone has been shown to activate hypoxia-inducible factor-1 (HIF-1) [13] and induce cell cycle arrest at the G1 phase through upregulation of the growth arrest and DNA damage-inducible gene 153 (CHOP/GADD153) in prostate cancer cells [14]. Recently, we demonstrated that ilimaquinone and its derivative, ethylsmenoquinone, inhibited the proliferation of multiple myeloma cells by downregulating intracellular β-catenin [15].

In this study, we used genetically-engineered HCT116 reporter cells to identify that ilimaquinone and ethylsmenoquinone activate p53-mediated transcriptional activity through stabilization of the p53 protein. We further demonstrated that both compounds induce G2/M cell cycle arrest, apoptosis and autophagy, thereby exhibiting anti-proliferative activity in colon cancer cells with the wild-type p53 gene.

## 2. Results and Discussion

### 2.1. Identification of Ilimaquinone and Ethylsmenoquinone as Activators of the p53 Pathway

**Figure 1:**
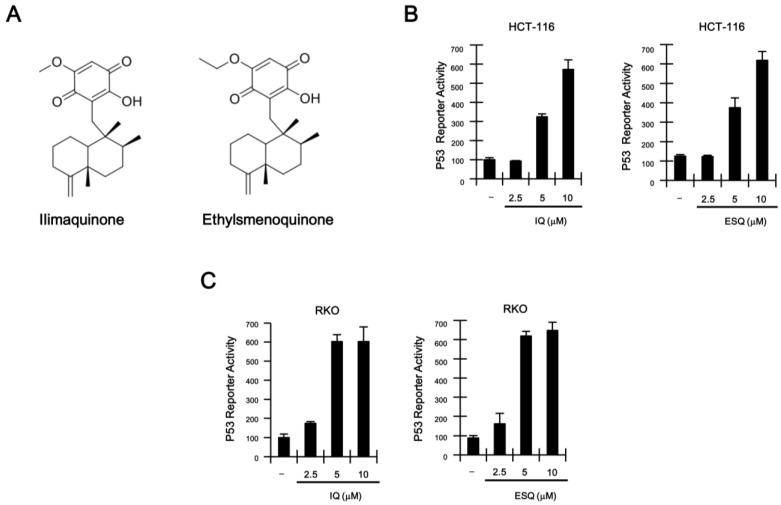
(**A**) Chemical structure of ilimaquinone (IQ) and ethylsmenoquinone (ESQ); (**B**,**C**) concentration-dependent activation of p53 response transcription by IQ and ESQ. HCT116-p53 FL and RKO-p53 FL reporter cells were incubated with the indicated concentrations of IQ and ESQ. After 15 h, firefly luciferase activity was determined. The results represent the average of three experiments. Bars indicate standard deviations.

To identify natural product-derived activators of the p53 signaling pathway, we stably transfected a synthetic p53-dependent luciferase reporter plasmid into HCT116 colon cancer cells, which harbor the wild-type p53 gene [16], thus generating HCT116-p53 firefly luciferase (FL) reporter cells. Screening of natural compounds with the HCT116-p53 FL reporter cells revealed that ilimaquinone and ethylsmenoquinone robustly activate p53 responsive transcription (Supplementary Figure S1 and Figure 1A; Supplementary Table 1). Treatment of HCT116-FL reporter cells with increasing concentrations of ilimaquinone and ethylsmenoquinone caused a dose-dependent activation of p53 responsive transcription (Figure 1C). Consistent with this result, we found that p53-driven FL activity was similarly upregulated by both compounds in RKO colon cancer cells (Figure 1D), which also express wild-type p53 [17].

**Figure 2:**
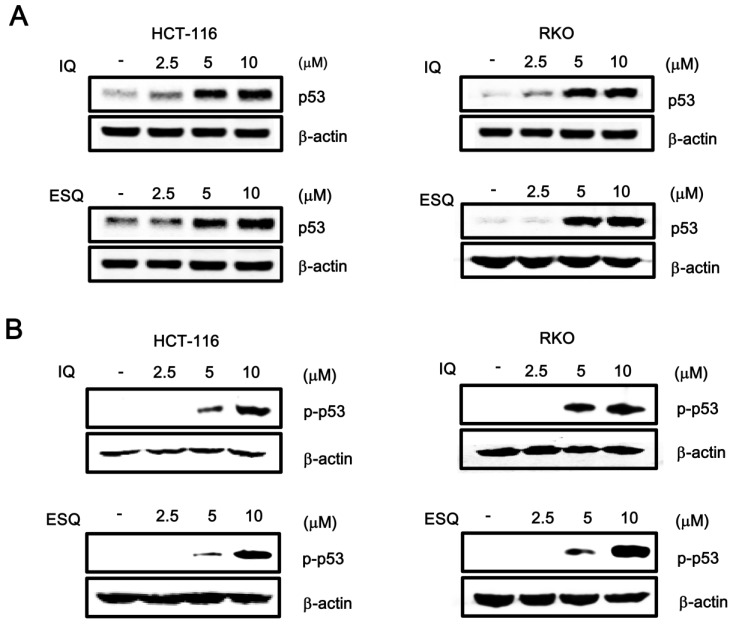
Ilimaquinone and ethylsmenoquinone promote the p53 and p-p53 level. (**A**,**B**) Whole proteins were prepared from HCT116 and RKO cells treated with vehicle (DMSO) or the indicated concentrations of IQ and ESQ for 15 h before being subjected to western blotting using anti-p53 and anti-p-p53 antibodies. Results are representative of three independent experiments.

Since p53 responsive transcription is dependent on the amount of intracellular p53, we investigated the effect of ilimaquinone and ethylsmenoquinone on p53 protein levels by western blot analysis using anti-p53 antibody. As shown in Figure 2A, incubation of either HCT116 or RKO cells with ilimaquinone and ethylsmenoquinone resulted in an increase in p53 protein, which is consistent with the p53-dependent reporter activity. Previous literature indicated that phosphorylation of p53 at the Ser15 residue inhibits the interaction of p53 with MDM2, as well as its subsequent ubiquitin-dependent degradation, thereby leading to p53 accumulation [6]. We thus performed western blot analysis with phospho-specific p53 antibody to test the effect of ilimaquinone and ethylsmenoquinone on this phosphorylation event. As expected, the addition of both compounds stimulated p53 phosphorylation at Ser15 in both HCT116 and RKO cells (Figure 2B), indicating that this phosphorylation is the mechanism behind ilimaquinone and ethylsmenoquinone-induced p53 stabilization and activation. We next tested the effects of phosphoinositide3 (PI3) kinases and AMP-activated kinase (AMPK), each of which catalyze the phosphorylation of p53 at Ser15, on ilimaquinone and ethylsmenoquinone-mediated activation of the p53 pathway. As shown in Supplementary Figure S2, caffeine and Ara-a, inhibitors of PI3 kinases and AMPK, respectively, did not affect the p53 responsive transcription induced by both compounds in HCT116 cells.

**Figure 3:**
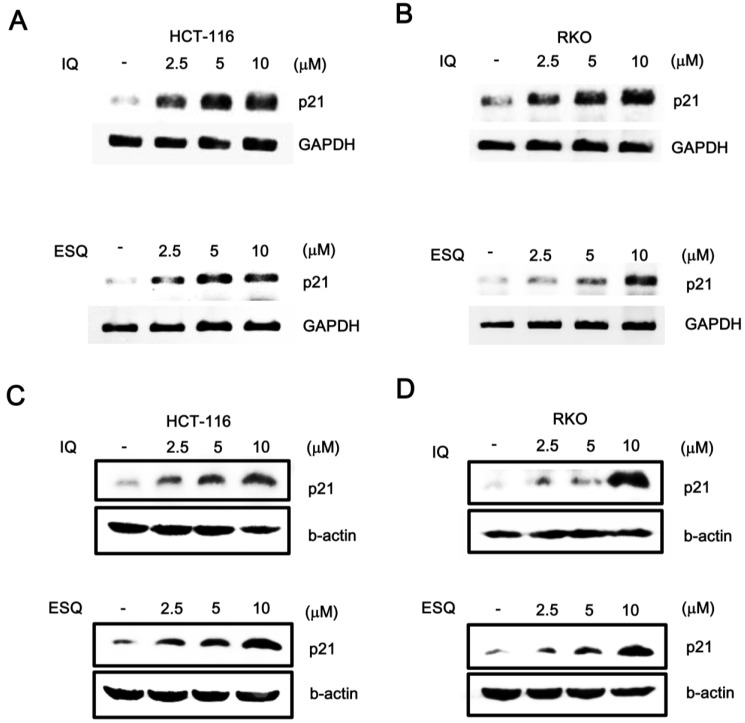
The effects of ilimaquinone and ethylsmenoquinone on the level of p21*^WAF1^*^/*CIP1*^ in colon cancer cells. (**A**,**B**) Semi-quantitative RT-PCR for p21*^WAF1^*^/*CIP1*^ and GAPDH were performed using total RNA prepared from HCT116 and RKO cells that were incubated with vehicle (DMSO) or the indicated concentrations of IQ and ESQ; the results are representative of three independent experiments; (**C**,**D**) Cell extracts from HCT116 and RKO cells treated with either vehicle (DMSO) or IQ and ESQ were analyzed by western blotting with anti-p21*^WAF1^*^/*CIP1*^ antibodies; In C and D, the blots were re-probed with anti-actin antibodies as a loading control. Results are representative of three independent experiments.

We next tested the effects of ilimaquinone and ethylsmenoquinone on the p53-dependent gene expression in colon cancer cells. A semi-quantitative RT-PCR assay demonstrated that the mRNA levels of p21*^WAF1^*^/*CIP1*^, a known target gene of p53, were elevated by treatment with ilimaquinone and ethylsmenoquinone in both HCT116 and RKO cells (Figure 3A,B). In conjunction with the resulting data, we observed that treatment with ilimaquinone and ethylsmenoquinone led to an increase in the level of p21*^WAF1^*^/*CIP1*^ protein (Figure 3C,D). Taken together, these results suggest that ilimaquinone and ethylsmenoquinone are specific activators of the p53 pathway.

### 2.2. Ilimaquinone and Ethylsmenoquinone Induce G_2_/M Cell Cycle Arrest in Colon Cancer Cells

**Figure 4:**
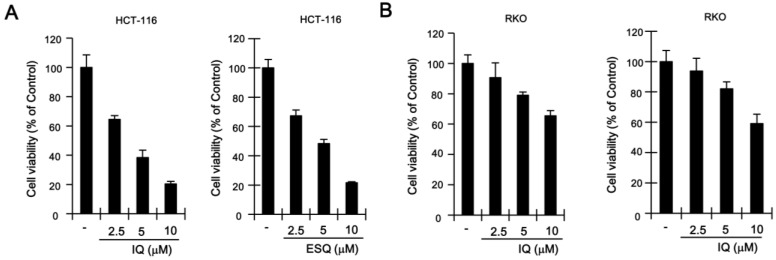

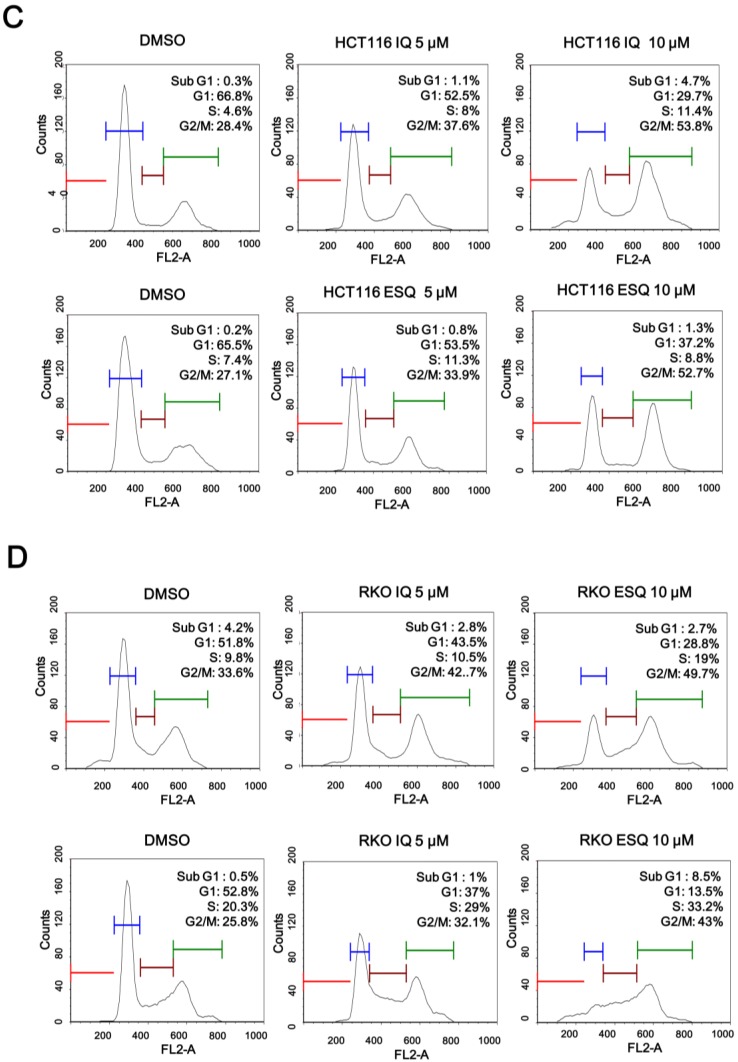
The effect of ilimaquinone and ethylsmenoquinone on colon cancer cell growth. (**A**,**B**) HCT116 (**A**) and RKO (**B**) cells were incubated for 24 h with the indicated concentrations of IQ and ESQ. Cell viability was examined using the CellTiter-Glo assay (Promega); (**C**,**D**) HCT116 (**C**) and RKO (**D**) cells were incubated with the vehicle (DMSO) or IQ and ESQ for 24 h. After incubation, cells were harvested and stained with propidium iodide (PI) and analyzed using a cytometer. The x-axis indicates the PI fluorescence intensity that correlates with the DNA content.

Previous studies have reported that p53, which is stabilized by certain anti-cancer drugs (e.g., doxorubicin and camptothecin), suppresses the proliferation of various cancer cells [18]. In light of the fact that ilimaquinone and ethylsmenoquinone induce the accumulation of p53, we postulated that these sesquiterpene quinones may also inhibit the growth of colon cancer cells. As expected, ilimaquinone and ethylsmenoquinone efficiently reduced the proliferation of both HCT116 and RKO cancer cells in a concentration-dependent manner (Figure 4A,B). To investigate the possible mechanism for ilimaquinone and ethylsmenoquinone-induced growth inhibition, cell cycle distribution after cell exposure to these compounds was analyzed using propidium iodide (PI) staining. When HCT116 were incubated with ilimaquinone and ethylsmenoquinone, the populations of cells in the G2/M phase increased from 28.4% to 53.8% and 27.1% to 52.7%, respectively. Similarly, when RKO cells were treated with ilimaquinone and ethylsmenoquinone, the G2/M phase cell populations increased from 33.6% to 49.7% and from 25.8% to 43%, respectively, compared with the vehicle control (Figure 4C,D). These results indicate that ilimaquinone and ethylsmenoquinone suppress the growth of colon cancer cells expressing wild-type p53 by inducing G2/M cell cycle arrest.

### 2.3. Ilimaquinone and Ethylsmenoquinone Induce Apoptosis in Colon Cancer Cells

**Figure 5:**
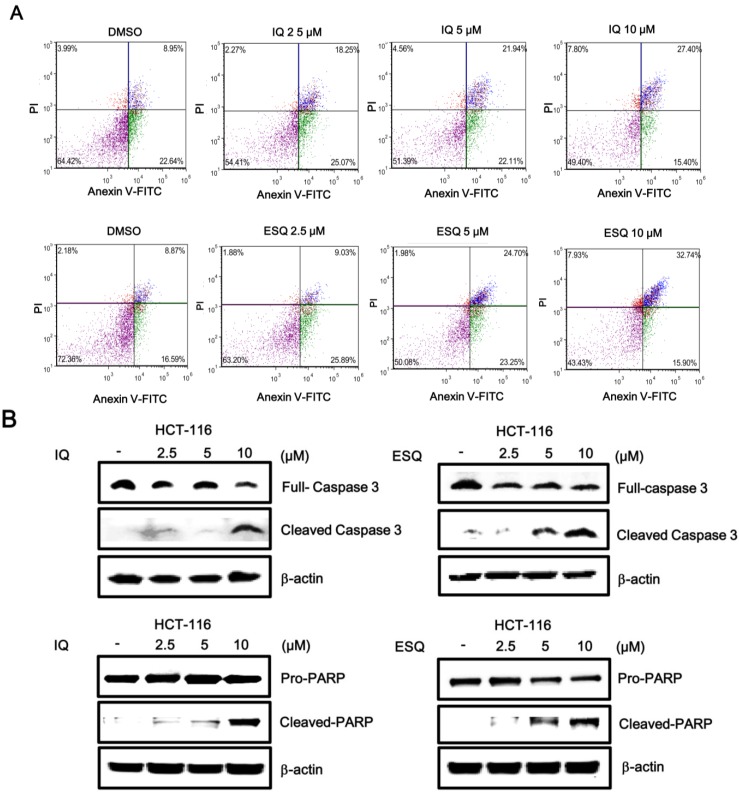
Ilimaquinone and ethylsmenoquinone induce apoptosis in HCT116 cells. (**A**) HCT116 cells were incubated with the vehicle (DMSO) or either IQ or ESQ for 24 h. Following treatment, cells were harvested and stained with Annexin V-FITC and PI and analyzed using a flow cytometry. The x-axis indicates the Annexin V-FITC intensity, while the y-axis indicates the PI fluorescence; (**B**) Cell extracts from HCT116 cells treated with either vehicle (DMSO) or the indicated concentrations of IQ and ESQ for 24 h were analyzed by western blotting with anti-caspase 3 and anti-PARP antibodies. The blots were re-probed with anti-actin antibodies as a loading control. Results are representative of three independent experiments.

Activation of the p53 pathway has been reported to induce apoptosis in various cancer cells [1,2]. Thus, we examined the abilities of ilimaquinone and ethylsmenoquinone to stimulate apoptosis in colon cancer cells. For this purpose, HCT116 cells were incubated with the sesquiterpene quinones, and the number of apoptotic cells was quantified using Annexin V/PI staining. As shown in Figure 5A, cytometric analysis revealed that the percentage of apoptotic cells significantly increased in a concentration-dependent manner. Compared to control cells, the percentage of Annexin V/PI double-positive cells increased from 8.95% to 27.4% and from 8.87% to 32.74% in HCT116 cells treated with 10 μM ilimaquinone and ethylsmenoquinone, respectively (Figure 5A). In addition, western blot analysis showed that treating HCT116 cells with either compound enhanced the proteolytic cleavage of pro-caspase-3, thereby activating caspase-3, which, in turn, catalyzed the cleavage of poly ADP ribose polymerase (PARP), a biochemical marker of apoptosis, (Figure 5B). This suggests that apoptosis contributed to ilimaquinone- and ethylsmenoquinone-induced cell death.

### 2.4. Ilimaquinone and Ethylsmenoquinone Induce Autophagy in Colon Cancer Cells

**Figure 6:**
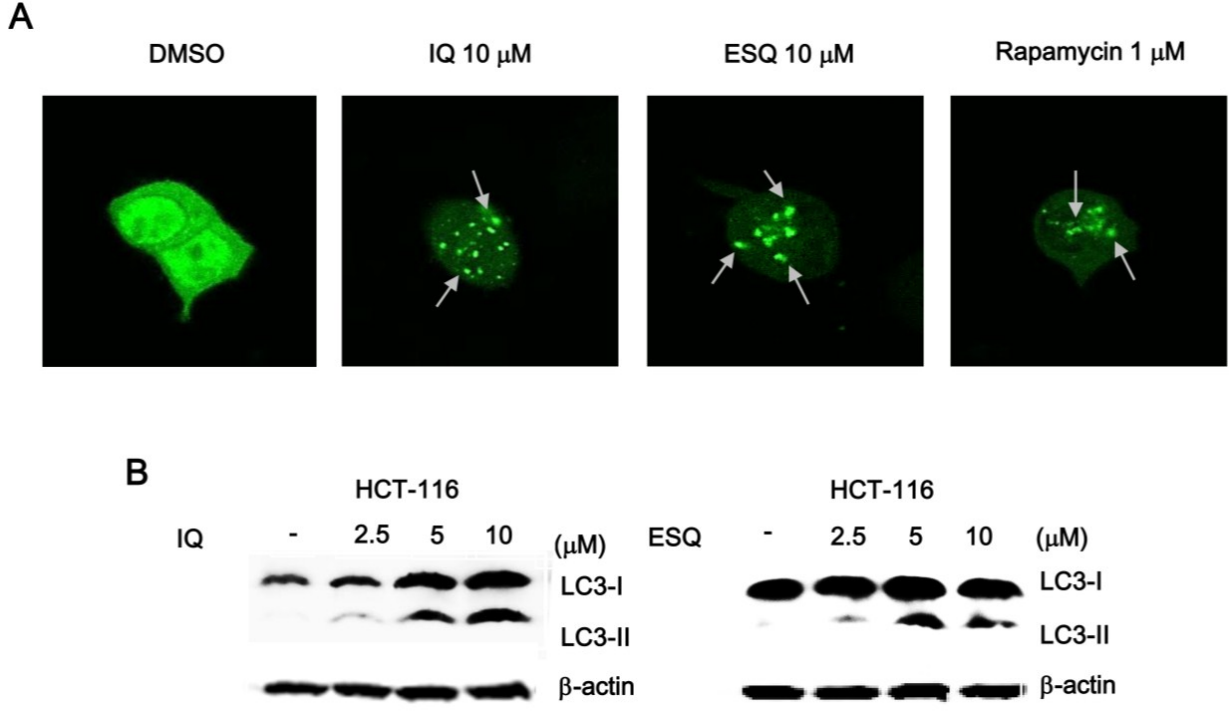
Ilimaquinone and ethylsmenoquinone induce autophagy in HCT116 cells. (**A**) Representative images of the punctate distribution of GFP-LC3-labeled in HCT-116 cells 24 h after exposure to IQ and ESQ; (**B**) HCT116 cells were treated with IQ and ESQ for 24 h or DMSO for 24 h. Cell lysates were separated on 15% SDS-PAGE and analyzed by western blot using anti-LC3 antibody. Results are representative of three independent experiments.

Recent studies have demonstrated that nuclear p53 negatively regulates the process of autophagy, which is a conserved catabolic pathway, by mediating the turnover of intracellular organelles and protein complexes [19]. Given that ilimaquinone and ethylsmenoquinone activated p53 responsive transcription in the nucleus, we next investigated whether these compounds induced autophagic cell death in colon cancer cells. For this purpose, we measured the conversion of the cytosolic microtubule-associated protein 1 light chain 3-1 (LC3-1) to the autophagosome-associated LC-3II form, which is widely used as an indication of autophagy [20]. HCT116 colon cancer cells were transfected with a green fluorescent protein tag of LC3 (GFP-LC3), and then, the cells were treated with ilimaquinone and ethylsmenoquinone. As depicted in Figure 6A and Supplementary Figure S3, these sesquiterpene quinone compounds stimulated the redistribution of GFP-LC3 from a diffuse pattern to punctuate dots. Furthermore, western blot analysis of the cell lysates revealed that ilimaquinone and ethylsmenoquinone promoted the formation of lipidated LC-3II from LC3-I (Figure 6B). These results indicate that autophagy may be a mechanism by which ilimaquinone and ethylsmenoquinone induce cell death in colon cancer cells.

## 3. Discussion

Colorectal cancer is one of the most common cancers and a major cause of cancer-related deaths in western countries [21]. Current therapies for colorectal cancer have relied on conventional cytotoxic agents, which have limited effect and cause substantial damage to healthy cells. Thus, there is a strong demand for the development of novel colorectal cancer therapeutics, and the p53 pathway is an attractive therapeutic target for colorectal cancer, because p53 functions as the most efficient barrier to caner development and progression of its prominent role in cell cycle arrest and cell death [9,10]. Ilimaquinone has been reported to potentiate TNF-related apoptosis-inducing ligand (TRAIL)-mediated apoptosis through upregulation of death receptor 4 and 5 in colon cancer cells [11]. The present study is the first to identify that ilimaquinone and ethylsmenoquinone from a marine sponge drive the accumulation of the p53 protein, thereby activating the p53 signaling pathway and efficiently inhibiting the growth of human colon cancer cells in the absence of TRAIL.

The p53 pathway regulates a number of biological processes, including DNA repair, metabolism, stemness, development, inflammation, endocytosis and cell death in response to a variety of cellular stressors [22]. Central to this pathway is the level of intracellular p53, which is controlled by its phosphorylation at Ser15 [7,8]. Several kinases have been reported to phosphorylate the Ser15 residue of p53. The ataxia-telangiectasia-mutated (ATM) kinase predominantly catalyzes p53 phosphorylation at this residue in response to genotoxic stresses, such as ionizing radiation or UV irradiation [23]. In addition, both extracellular signal-regulated kinase 1/2 (ERK1/2)- and p38 mitogen-activated protein kinase (p38 MAPK)-mediated p53 phosphorylation of Ser15 stabilize intracellular p53 protein levels in several human cancer cell types [24,25]. Furthermore, AMP-activated protein kinase (AMPK) also catalyzes the stabilizing phosphorylation of p53 at Ser15 [26]. In the present study, pharmacological inhibitors of PI3 kinases (ATM, ERK1/2 and p38) and AMPK did not affect ilimaquinone and ethylsmenoquinone-induced p53 activation, indicating that these kinases are not required for the agonistic activity of ilimaquinone and ethylsmenoquinone within the p53 pathway. The mechanism underlying ilimaquinone- and ethylsmenoquinone-induced p53 phosphorylation and stabilization needs to be investigated further.

Aberrant upregulation of β-catenin levels is involved in the development and progression of colorectal cancer [27]. Truncated forms of adenomatous polyposis coli (APC), a component of the β-catenin destruction complex [28], are observed in the majority of sporadic colorectal cancer cases [29]. In addition, the N-terminal phosphorylation motif of β-catenin is frequently mutated in colorectal cancer [30]. A recent study has reported that ilimaquinone and ethylsmenoquinone promote β-catenin degradation in multiple myeloma cells [15], which exhibit β-catenin overexpression without activating mutations of the Wnt/β-catenin pathway [31]. In this study, ilimaquinone and ethylsmenoquinone did not decrease the intracellular β-catenin level in HCT116 colon cancer cells that contain a Ser45 deletion mutation in β-catenin (Supplementary Figure S4), suggesting that inhibition of the Wnt/β-catenin pathway is not the responsible mechanism by which ilimaquinone and ethylsmenoquinone suppress the proliferation of colon cancer cells.

It has been reported that p21*^WAF1^*^/*CIP1*^, a well-established target gene of the p53 pathway, controls both the G_2_/M and G_1_ cell cycle check points [32]. p21*^WAF1^*^/*CIP1*^ forms a complex with G_2_/M-specific cdc2 kinase, thereby inhibiting cdc2 kinase activity and inducing G_2_/M cell cycle arrest. In addition, p21*^WAF1^*^/*CIP1*^ also plays an important role in the maintenance of G_2_/M cell cycle arrest by blocking the interaction of cdc25C with proliferating cell nuclear antigen (PCNA) [33]. In the present study, ilimaquinone and ethylsmenoquinone stabilized p53 through Ser15 phosphorylation and, thus, upregulated p21*^WAF1^*^/*CIP1*^ expression, leading to induction of G_2_/M cell cycle arrest in colon cancer cells.

Apoptosis is a conserved programmed cell death mechanism and is involved in the elimination of damaged or cancerous cells [34]. Many anti-cancer agents induce the intrinsic apoptotic pathway, which is characterized by increases in the Bax/Bcl2 ratio, the activation of caspases and cleavage of PARP [35]. Autophagy functions as a cellular homeostatic mechanism through the autophagosome and lysosome system in response to cellular stress [19]. Autophagy plays a quite complex role in cancer development and can exert either a tumor-progressive or a tumor-suppressive effect [36]. Apoptosis and autophagy may be interconnected, either antagonistically or cooperatively, in response to various anti-cancer therapeutics in different cancer cells [37]. Furthermore, p53 has been demonstrated to simultaneously control both apoptosis and autophagy by upregulating apoptotic genes and regulating multiple levels of the AMPK-mTOR axis, respectively [9]. We observed that the activation of caspase-3, an executor of apoptosis, and the formation of LC-3II, a marker of autophagy, were induced by ilimaquinone and ethylsmenoquinone, which were identified as activators of the p53 pathway in colon cancer cells, thereby demonstrating that ilimaquinone and ethylsmenoquinone induce both apoptosis and autophagy-mediated cell death.

## 4. Experimental Section

### 4.1. Isolation of Ilimaquinone and Synthesis of Ethylsmenoquinone

Ilimaquinone was obtained from our previous chemical investigation of three sponges, *Smenospongia aurea*, *S. cerebriformis* and *Verongula rigida* [38]. Ethylsmenoquinone was synthesized as previously described [15].

### 4.2. Cell Culture, Transfection and Luciferase Assay

HCT116 and RKO cells were obtained from the American Type Culture Collection and maintained in DMEM supplemented with 10% fetal bovine serum (FBS), 120 μg/mL penicillin and 200 μg/mL streptomycin. HCT116-p53 FL reporter cells and RKO-p53 FL reporter cells were established as previously described [39]. Transfections were performed using Lipofectamine 2000 (Invitrogen, Grand Island, NY, USA) according to the manufacturer’s instruction. The luciferase assay was performed using the Dual Luciferase Assay Kit (Promega, Madison, WI, USA).

### 4.3. Screening for Small Molecule Activators of the p53 Pathway

HCT116-p53-FL reporter cells were inoculated into 96-well plates at a density of 15,000 cells per well and grown for 24 h. The cells were incubated with natural compounds at a final concentration of 20 μM for 15 h and the plates assayed for firefly luciferase activity and cell viability.

### 4.4. Western Blot Analysis

Proteins were separated by SDS-PAGE in a 4%–12% gradient gel (Invitrogen, Carlsbad, CA, USA) and transferred to nitrocellulose membranes (Bio-Rad, Hercules, CA, USA). The membranes were blocked with 5% nonfat milk and probed with anti-p53 (Santa Cruz Biotechnology, Santa Cruz, CA, USA), anti-p-p53 (Santa Cruz Biotechnology), anti-p21 (Santa Cruz Biotechnology), anti-PARP (Cell Signaling Technology, Denvers, MA, USA), anti-cleaved caspase 3 (Cell Signaling Technology), anti-LC3B (Cell Signaling Technology) and anti-actin antibodies (Cell Signaling Technology). The membranes were then incubated with horseradish-peroxidase-conjugated anti-mouse IgG (Santa Cruz Biotechnology) or anti-rabbit IgG (Santa Cruz Biotechnology) and visualized using the ECL system (Santa Cruz Biotechnology).

### 4.5. RNA Extraction and Semi-Quantitative RT-PCR

Total RNA was isolated with TRIzol reagent (Invitrogen, Carlsbad, CA, USA) in accordance with the manufacturer’s instructions. Synthesis of cDNA, reverse transcription and PCR were performed as previously described [40]. The amplified DNA was separated on 2% agarose gels and stained with ethidium bromide.

### 4.6. Cell Viability Assay

Cells were seeded into 96-well plates and treated with ilimaquinone and ethylsmenoquinone for 24 h. Cell viability from each treated sample was measured in triplicate by using the Celltiter-Glo assay kit (Promega, Madison, WI, USA) according to the manufacturer’s instructions.

### 4.7. Cell Cycle Analysis

Cells were treated with ilimaquinone and ethylsmenoquinone for 24 h. Cells were then collected, washed with cold phosphate-buffered saline (PBS) and fixed in 70% ethanol at 4 °C overnight. They were then centrifuged at 2000 rpm for 5 min, resuspended in PBS and incubated with propidium iodide (100 μg/mL) and RNase A (50 μg/mL) at room temperature for 30 min in the dark. Cells were then analyzed using a Cellometer cytometer (Nexcelom Bioscience, Lawrence, MA, USA).

### 4.8. Apoptosis Analysis

After cells were treated with ilimaquinone and ethylsmenoquinone for 24 h, cells were washed with cold PBS and resuspended in staining buffer containing Annexin V-FITC and propidium iodide, provided in the apoptosis detection kit (BD Transduction Laboratories), according to the manufacturer’s instructions. Cells were then analyzed using a Cellometer cytometer (Nexcelom, Lawrence, MA, USA).

### 4.9. Confocal Microscopy of GFP-LC3 Fluorescence

GFP-LC3-transfected cells were incubated with ilimaquinone and ethylsmenoquinone for 15 h and fixed with 4% paraformaldehyde in PBS at room temperature. Fluorescence signals were visualized and captured by a Leica 4000 confocal microscope (Leica Microsystems, Mannheim, Germany).

## 5. Conclusions

We investigated the anticancer effect of ilimaquinone and ethylsmenoquinone on colon cancer cells and revealed their mechanism of action using a cell-based reporter system. Ilimaquinone and ethylsmenoquinone activate the p53 pathway by promoting the phosphorylation and accumulation of the p53 protein, in turn inhibiting the proliferation of colon cancer cells by inducing G_2_/M cell cycle arrest, apoptosis and autophagy. Therefore, ilimaquinone and ethylsmenoquinone have the potential to be effective cancer preventive agents and anti-neoplastic therapeutics for colorectal cancer.

## Supplementary Files

Supplementary File 1
