# Supplementary material for: Activation of p53 with Ilimaquinone and Ethylsmenoquinone, Marine Sponge Metabolites, Induces Apoptosis and Autophagy in Colon Cancer Cells
Source: Mar Drugs. 2015 Jan 16;13(1):543–57. doi: 10.3390/md13010543 (PMC4306951; doi:10.3390/md13010543)
Supplement: Supplementary File 1 [file marinedrugs-13-00543-s001.pdf]

Supplementary Information

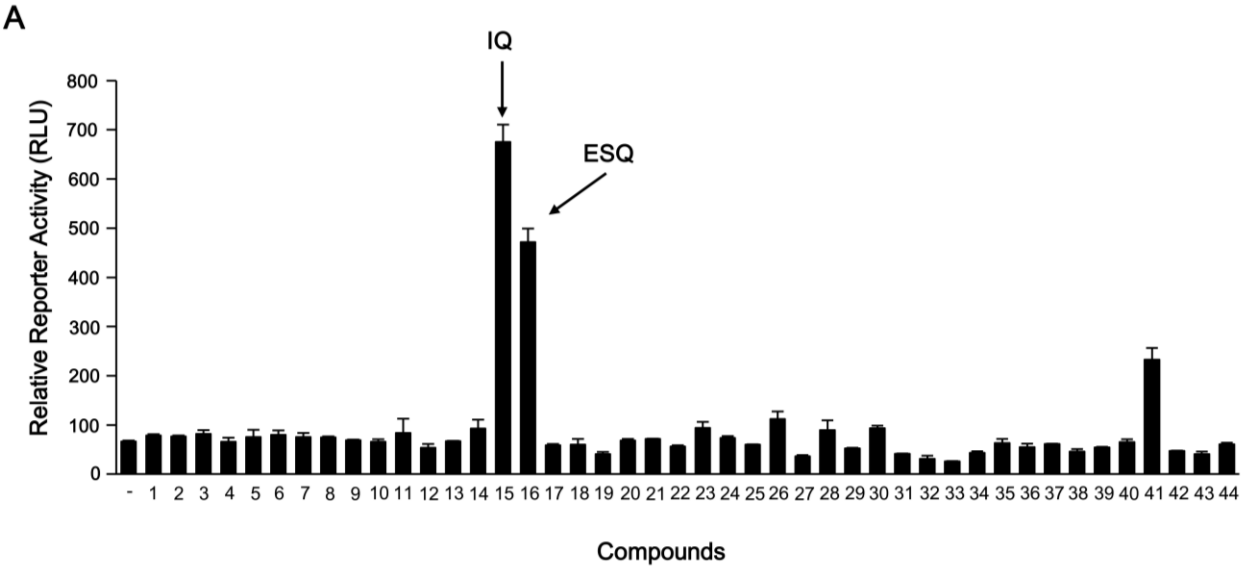

Figure S1. Screening of compounds that activate the p53 pathway. HCT116-p53 FL reporter cells were incubated with natural compounds. After 15 h, firefly luciferase activity was determined. The results represent the average of three experiments.

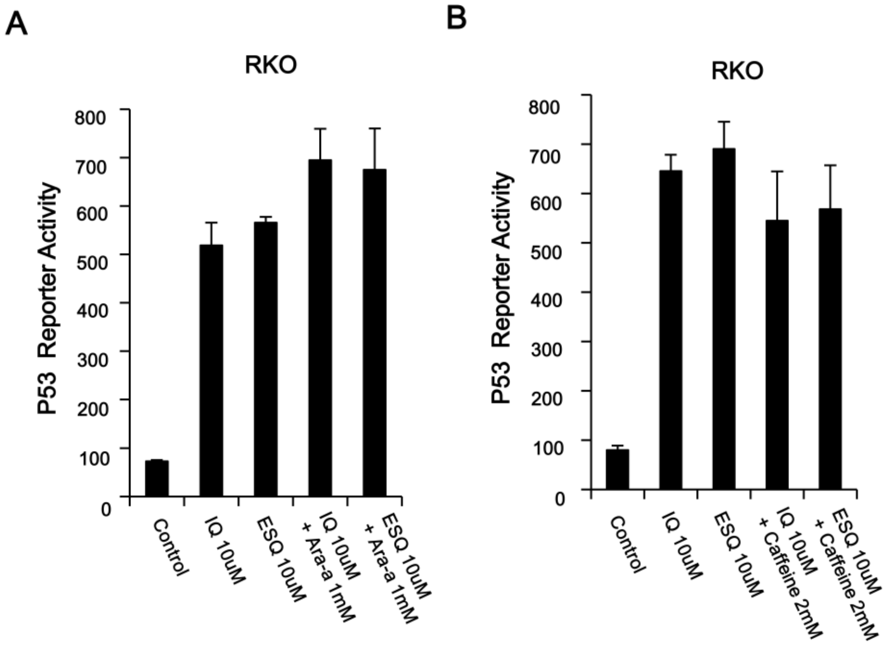

Figure S2. Pharmaological inhibitors of ATM and AMPK did not abrogate ilimaquinone- and ethylsmenoquinone-mediated p53 activation. (**A**,**B**) RKO-p53 FL reporter cells were treated with IQ (10 μM) or ESQ (10 μM) with or without caffeine (2 Mm) (**A**) or compound C (10 μM) (**B**). After 15 h, firefly luciferase activity was determined. The results represent the average of three experiments. Bars indicate standard deciations.

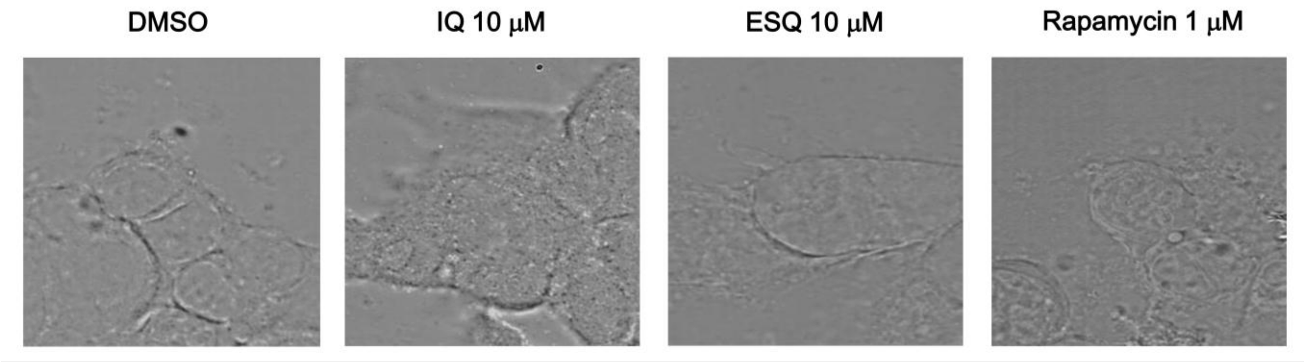

Figure S3. Representative light field images of GFP-LC3-labeled in HCT-116 cells 24 h after exposure to IQ and ESQ.

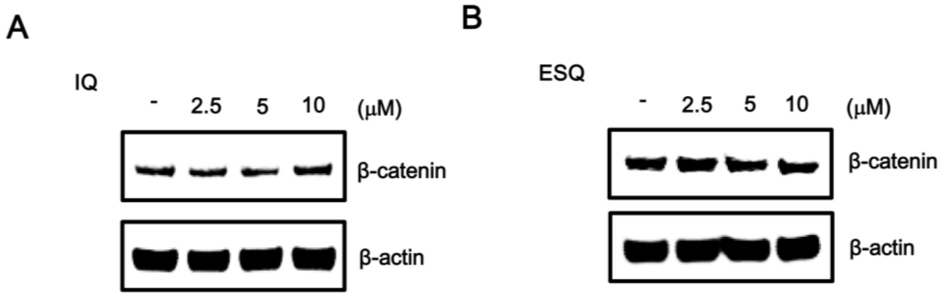

Figure S4. ilimaquinone and ethylsmenoquinone did not affect on the intracellular β-catenin level in HCT116 cells. (**A**,**B**) Cytosolic proteins were prepared from HCT116 cells treated with vehicle (DMSO) or the indicated concentrations of IQ and ESQ for 15 h before being subjected to western blotting using anti-β-catenin antibodies. The blots were re-probed with anti-actin antibodies as a loading control.

Table S1. The natural compounds used in this study.

	Compound
**1**	3,5-dibromo-1-hydroxy-4,4-dimethoxy-2,5-cyclohexadiene-1-acetamide
**2**	Aeroplysinin-2
**3**	Aeroplysinin-1
**4**	2-(3,5-dibromo-2-hydroxy-4-methoxyphenyl)acetic acid
**5**	*Z*-3,5-dibromo-4-ethoxy-1-hydroxy-4-methoxycyclohexa-2,5-dien-1-yl acetic acid
**6**	2-(3,5-dibromo-4-hydroxyphenyl)acetamide
**7**	3-(3-bromo-4-hydroxyphenyl)propanoic acid
**8**	purealidin R
**9**	2-(3,5-Dibromo-4,4-diethoxy-1-hydroxycyclohexa-2,5-dien-1-yl)acetamide
**10**	(*R*,*S*)-[3.5-dibromo-4-[(2-oxo-5-oxazolidinyl)]methoxyphenyl]-2-oxazolidinone
**11**	smenospongidine
**12**	Dactyloquinone B
**13**	Dactyloquinone D
**14**	Cyclospongiaquinone-1
**15**	ilimaquinone

Table S1. *Cont.*

**16**	5-epi-ilimaquinone
**17**	(−)-Nakijinol E
**18**	(+)-5-epi-nakijinol E
**19**	Mixture of nakijinone A and 5-epi-nakijinone A
**20**	Thimine
**21**	L-thymidine
**22**	(−)-Dactylospongenone E
**23**	Mixture of 5-epi-dactylospongenone E and 5-epi-dactylospongenone F
**24**	Debromoaplysin
**25**	Debromoisolaurinterol
**26**	Debromolaurinterol
**27**	Laurinterol
**28**	Debromolaurinterol acetate
**29**	Epoxyvenustin
**30**	15-bromo-2,16-diacetoxyparguer-9(11)-en-7,16-diol
**31**	Isodictytriol
**32**	Aplykurodin A
**33**	Aplykurodin B
**34**	(±)-Aplysin 20
**35**	Desmosterol
**36**	Cholesterol
**37**	3β,5β,14α-trihydroxy cholest-7-en-6-one
**38**	5α,6α-Epoxycholest-8(14)-ene-3β,7α-diol
**39**	Cholest-7-ene-3β,5α,6β-triol
**40**	α-Tocopherolquinol
**41**	Protopine
**42**	Nantenine
**43**	Nornantenine
**44**	Tetrahydroberberrubine

© 2015 by the authors; licensee MDPI, Basel, Switzerland. This article is an open access article distributed under the terms and conditions of the Creative Commons Attribution license (http://creativecommons.org/licenses/by/4.0/).
